# Early neurological deterioration after intravenous thrombolysis of anterior vs posterior circulation stroke: a secondary analysis of INTRECIS

**DOI:** 10.1038/s41598-022-07095-6

**Published:** 2022-02-24

**Authors:** Yu Cui, Wei-Hong Meng, Hui-Sheng Chen

**Affiliations:** 1 Department of Life Science and Biopharmaceutics, Shenyang Pharmaceutical University, 103 Wenhua Rd, Shenyang, 110016 China; 2 Department of Neurology, General Hospital of Northern Theater Command, 83 Wenhua Rd, Shenyang, 110016 China

**Keywords:** Neuroscience, Neurology

## Abstract

Anterior circulation stroke (ACS) differs from posterior circulation stroke (PCS) in many ways, but it remains unclear whether there is any difference in early neurological deterioration (END) in two stroke territories. We compared post-thrombolytic END between ACS and PCS based on the data from INTRECIS. We screened patients receiving intravenous 0.9 mg/kg alteplase within 4.5 h in the INTRECIS cohort. According to stroke territory, patients were divided into ACS and PCS groups. The primary outcome was incidence of END, which was defined as an increase in NIHSS score ≥ 4 or death within 24 h from baseline. The secondary outcomes were associated factors of END and 90-day modified Rankin Scale (mRS) distribution. Overall, 1194 patients were enrolled in this study: 942 in ACS group and 252 in PCS group. There was no significant difference in the incidence of END between two groups (3.8% vs 5.2%, adjusted *p* = 0.406). Atrial fibrillation (adjusted *p* = 0.012) and TOAST classification (adjusted *p* = 0.009) were associated with END in ACS, while hypertension history (adjusted *p* = 0.046) and baseline NIHSS score (adjusted *p* = 0.011) with END in PCS. END was associated with worse outcome on 90-day mRS in ACS and PCS (adjusted *p* < 0.001). Based on a prospective nationwide cohort, we provided first report for similar incidence, but different risk factors of post-thrombolytic END in ACS vs PCS patients.

**Trial Registration-URL**: https://www.clinicaltrials.gov; Unique identifier: NCT02854592.

## Introduction

According to vascular territories occurring ischemic lesions, ischemic stroke can be broadly divided into anterior circulation stroke (ACS) and posterior circulation stroke (PCS). Intravenous thrombolysis with alteplase is an effective treatment for acute ischemic stroke^1,2^.

Some previous studies showed different post-thrombolytic outcomes between ACS and PCS^3,4^, which may be attributed to the difference in etiology, symptoms, and risk factors^5^, while Sommer et al. did not find the different functional outcomes between PCS and ACS^6^.

Early neurological deterioration (END) occurring in 24 h after stroke is unpredictable and may influence the outcome for patients receiving intravenous thrombolysis^7^. Previous studies have comprehensively investigated the incidence, risk factors, and prognosis of END, however most of these studies focused on ACS^8,9^. Notably, up to date, no study has investigated whether there is difference in post-thrombolytic END between ACS and PCS.

INtravenous Thrombolysis REgistry for Chinese Ischaemic Stroke within 4.5 h of onset (INTRECIS) is a ‘real world’, prospective, nationwide, and multicenter registry study in China^10^. Based on the data from INTRECIS study, we aimed to compare the incidence of post-thrombolytic END between ACS and PCS. Furthermore, we investigated potential associated factors of END in ACS and PCS, respectively.

## Results

**Figure 1:**
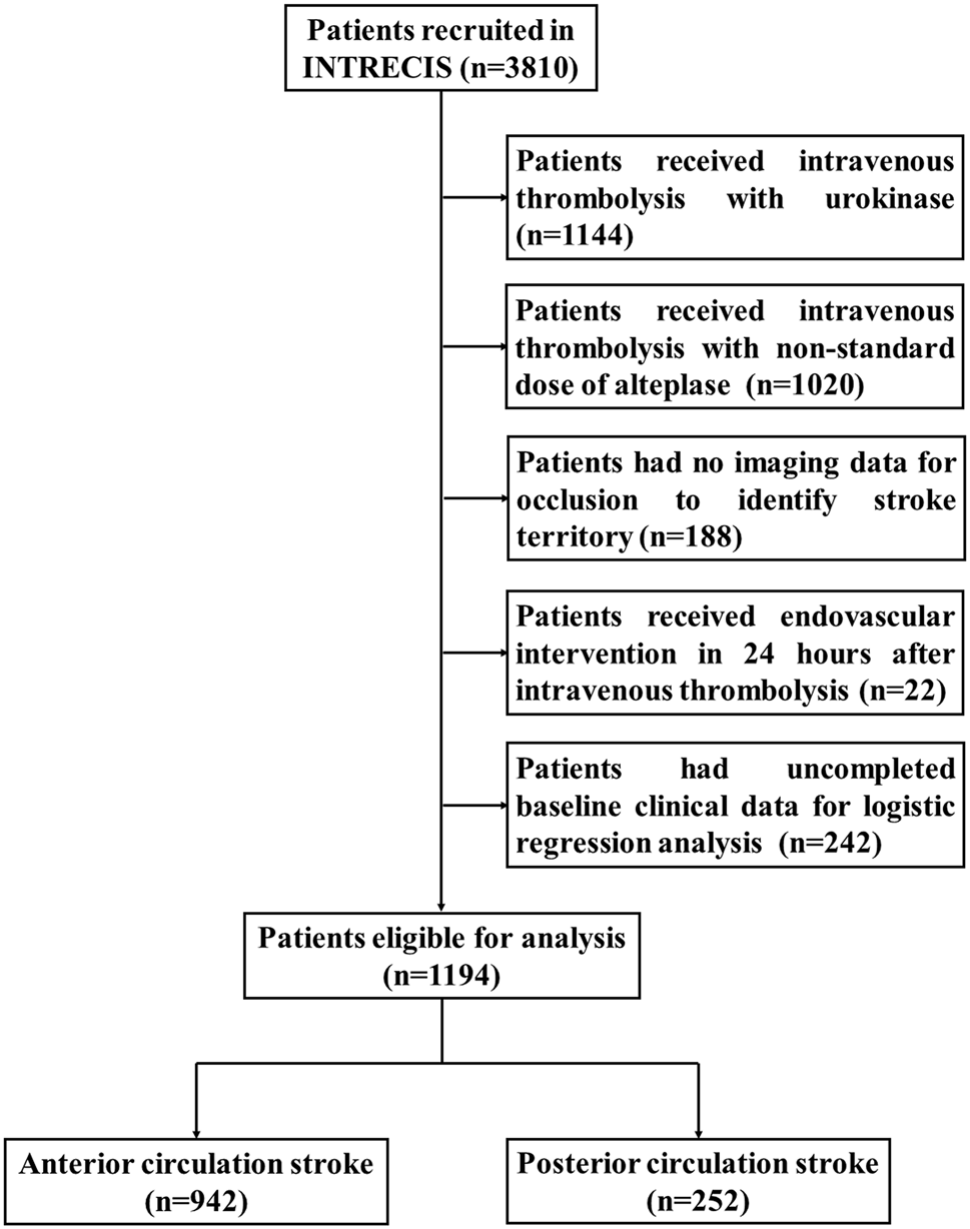
Flow chart of eligible patients. *INTRECIS* intravenous thrombolysis registry for Chinese Ischaemic stroke within 4.5 h of onset.

**Figure 2:**
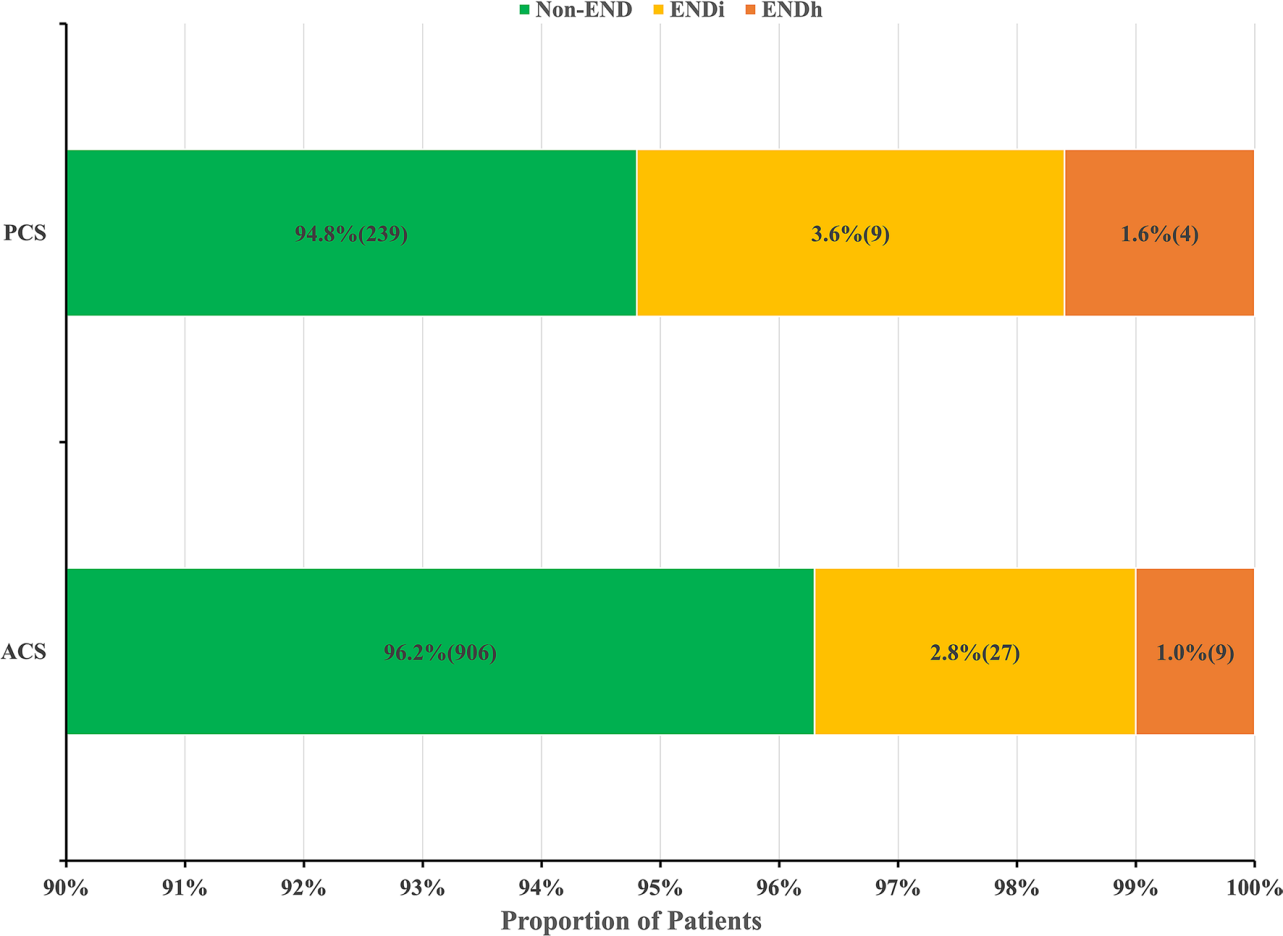
Proportion of patients in anterior vs posterior circulation stroke groups. *ACS* anterior circulation stroke, *END* early neurological deterioration, *ENDi* END due to ischemic injure; *ENDh* END due to hemorrhagic injure, *PCS* posterior circulation stroke.

Out of 3810 patients enrolled in INTRECIS cohort between April 2017 and July 2019, 1194 patients were included in the present study: 942 (78.9%) in ACS group and 252 (21.1%) in PCS group (Fig. 1). Baseline characteristics in two groups were shown in Table 1. Patients with PCS had more hypertension (51.6% vs 65.9%) and diabetes mellitus history (16.2% vs 27.4%), higher body mass index (23.8 kg/m^2^ vs 24.5 kg/m^2^) and blood glucose (6.80 mmol/L vs 7.30 mmol/L), longer symptom onset to thrombolysis time (165 min vs 179 min), and less atrial fibrillation (10.6% vs 5.6%) and cardioembolism (14.8% vs 7.1%). END occurred in 49 (4.1%) patients: 36 (27 with ischemic END and 9 with hemorrhagic END) in ACS group and 13 (9 with ischemic END and 4 with hemorrhagic END) in PCS group (Fig. 2). After adjusting all the baseline variables, the incidence of END was similar in ACS group and PCS group (3.8% vs 5.2%, OR = 0.750, 95% CI 0.381–1.477, adjusted *p* = 0.406).

**Table 1:** Baseline characteristics in ACS group and PCS group.

Variable	ACS (n = 942)	PCS (n = 252)	*P* Value
Age (years), median (IQR)	64 (56–72)	62 (55–70)	0.066
Gender (male), n (%)	640 (67.9)	164 (65.1)	0.390
Current smoker, n (%)	365 (38.7)	95 (37.7)	0.761
Current drinker, n (%)	218 (23.1)	62 (24.6)	0.627
Hypertension, n (%)	486 (51.6)	166 (65.9)	0.000
Diabetes mellitus, n (%)	153 (16.2)	69 (27.4)	0.000
Hyperlipidemia, n (%)	36 (3.8)	10 (4.0)	0.914
Coronary heart disease, n (%)	132 (14.0)	36 (14.3)	0.912
Atrial fibrillation, n (%)	100 (10.6)	14 (5.6)	0.015
History of stroke, n (%)	157 (16.7)	54 (21.4)	0.078
BMI (kg/m^2^), median (IQR)	23.8 (21.1–26.1)	24.5 (22.0–27.1)	0.002
SBP (mmHg), median (IQR)	151 (137–165)	150 (134–169)	0.609
DBP (mmHg), median (IQR)	88 (80–98)	89 (80–99)	0.789
OTT (min), median (IQR)	165 (125–206)	179 (143–216)	0.016
DNT (min), median (IQR)	54 (34–85)	60 (37–85)	0.922
Baseline NIHSS, median (IQR)	6 (3–11)	5 (3–9)	0.904
BG (mmol/L), median (IQR)	6.80 (5.80–8.60)	7.30 (6.12–10.00)	0.001
**TOAST classification**			0.000
LAA, n (%)	470 (49.9)	133 (52.8)	
CE, n (%)	139 (14.8)	18 (7.1)	
SAO, n (%)	261 (27.7)	78 (31.0)	
ODC, n (%)	19 (2.0)	7 (2.8)	
UND, n (%)	53 (5.6)	16 (6.3)	

*ACS* anterior circulation stroke, *BG* blood glucose, *BMI* body mass index, *CE* cardioembolism, *DBP* diastolic blood pressure, *DNT* door to needle time, *IQR* interquartile range, *LAA* large-artery atherosclerosis, *NIHSS* National Institute of Health Stroke Scale, *ODC* stroke of other determined cause, *OTT* symptom onset to thrombolysis time, *PCS* posterior circulation stroke, *SAO* small-artery occlusion, *SBP* systolic blood pressure, *TOAST* trial of Org 10,172 in acute stroke treatment, *UND* stroke of undetermined cause.

Multivariate logistic regression analysis showed that the most significant independent factors associated with END were atrial fibrillation (OR = 3.657, 95% CI 1.323–10.107, adjusted *p* = 0.012) and TOAST classification (OR = 1.703, 95% CI 1.142–2.538, adjusted *p* = 0.009) in ACS group (Table 2), while hypertension history (OR = 11.298, 95% CI 1.043–122.409, adjusted *p* = 0.046) and baseline NIHSS score (OR = 1.099, 95% CI 1.021–1.182, adjusted *p* = 0.011) in PCS group (Table 3).

**Table 2:** Multivariate logistic regression analysis on associated factors in ACS group.

Variables	END (n = 36)	Non-END (n = 906)	OR	95% CI	Adjusted *P* value
Age (year), median (IQR)	63 (54–69)	64 (56–72)	0.974	0.941–1.007	0.125
Gender (male), n (%)	25 (69.4)	615 (67.9)	1.318	0.558–3.114	0.528
Current smoker, n (%)	12 (33.3)	353 (39.0)	0.582	0.234–1.444	0.243
Current drinker, n (%)	8 (22.2)	210 (23.2)	0.790	0.290–2.155	0.645
Hypertension, n (%)	22 (61.1)	464 (48.8)	1.843	0.864–3.931	0.114
Diabetes mellitus, n (%)	5 (13.9)	148 (16.3)	0.810	0.257–2.559	0.720
Hyperlipidemia, n (%)	0 (0.0)	36 (4.0)	0.000	0.000-	0.998
Coronary heart disease, n (%)	5 (13.9)	127 (14.0)	0.784	0.277–2.221	0.647
Atrial fibrillation, n (%)	7 (19.4)	93 (10.3)	3.657	1.323–10.107	0.012
History of stroke, n (%)	5 (13.9)	152 (16.8)	0.749	0.273–2.059	0.576
BMI (kg/m^2^), median (IQR)	23.8 (20.8–26.7)	23.8 (21.1–26.1)	0.970	0.876–1.073	0.551
SBP (mmHg), median (IQR)	151 (131–165)	151 (137–165)	1.001	0.981–1.022	0.932
DBP (mmHg), median (IQR)	90 (79–99)	88 (80–98)	1.018	0.986–1.051	0.273
OTT (min), median (IQR)	169 (123–213)	165 (125–206)	1.002	0.996–1.009	0.456
DNT (min), median (IQR)	58 (39–84)	54 (34–85)	1.003	0.995–1.011	0.425
Baseline NIHSS, median (IQR)	6 (2–9)	6 (3–11)	0.947	0.884–1.015	0.124
BG (mmol/L), median (IQR)	6.74 (5.87–7.96)	6.80 (5.80–8.61)	0.984	0.860–1.126	0.814
NLR at admission, median (IQR)	2.02 (1.37–5.17)	2.78 (1.80–4.63)	0.938	0.813–1.081	0.376
**TOAST classification**			1.703	1.142–2.538	0.009
LAA, n (%)	24 (66.7)	446 (49.2)			
CE, n (%)	4 (11.1)	135 (14.9)			
SAO, n (%)	8 (22.2)	253 (27.9)			
ODC, n (%)	0 (0.0)	19 (2.1)			
UND, n (%)	0 (0.0)	53 (5.8)			

*ACS* anterior circulation stroke, *BG* blood glucose, *BMI* body mass index, *CE* cardioembolism, *DBP* diastolic blood pressure, *DNT* door to needle time, *END* early neurological deterioration, *IQR* interquartile range, *LAA* large-artery atherosclerosis, *NIHSS* National Institute of Health Stroke Scale, *NLR* neutrophil-to-lymphocyte ratio, *ODC* stroke of other determined cause, *OTT* symptom onset to thrombolysis time, *SAO* small-artery occlusion, *SBP* systolic blood pressure, *TOAST* trial of Org 10,172 in acute stroke treatment, *UND* stroke of undetermined cause.

**Table 3:** Multivariate logistic regression analysis on associated factors in PCS group.

Variables	END (n = 13)	Non-END (n = 239)	OR	95% CI	Adjusted *P* value
Age (year), median (IQR)	66 (53–73)	62 (55–70)	1.026	0.959–1.096	0.457
Gender (male), n (%)	10 (76.9)	154 (64.4)	1.752	0.302–10.170	0.532
Current smoker, n (%)	6 (46.2)	89 (37.2)	1.048	0.205–5.346	0.955
Current drinker, n (%)	4 (30.8)	58 (24.3)	2.462	0.397–15.257	0.333
Hypertension, n (%)	12 (92.3)	154 (64.4)	11.298	1.043–122.409	0.046
Diabetes mellitus, n (%)	4 (30.8)	65 (27.2)	1.517	0.328–7.008	0.593
Hyperlipidemia, n (%)	0 (0.0)	10 (4.2)	0.000	0.000	0.998
Coronary heart disease, n (%)	2 (15.4)	34 (14.2)	1.079	0.189–6.157	0.932
Atrial fibrillation, n (%)	0 (0.0)	14 (5.9)	0.000	0.000	0.999
History of stroke, n (%)	5 (38.5)	49 (20.5)	2.375	0.615–9.172	0.210
BMI (kg/m^2^), median (IQR)	23.0 (21.5–27.6)	24.5 (22.0–27.1)	0.920	0.749–1.131	0.429
SBP (mmHg), median (IQR)	159 (134–182)	150 (134–169)	1.016	0.982–1.050	0.357
DBP (mmHg), median (IQR)	90 (80–99)	89 (80–99)	1.065	0.990–1.146	0.091
OTT (min), median (IQR)	175 (119–194)	180 (143–217)	0.993	0.981–1.005	0.239
DNT (min), median (IQR)	60 (31–102)	60 (37–85)	1.002	0.984–1.020	0.853
Baseline NIHSS, median (IQR)	10 (5–22)	5 (3–9)	1.099	1.021–1.182	0.011
BG (mmol/L), median (IQR)	8.29 (6.15–10.13)	7.30 (6.10–9.99)	1.044	0.881–1.238	0.617
NLR at admission, median (IQR)	7.46 (2.15–10.20)	6.33 (3.93–8.73)	1.037	0.998–1.078	0.062
**TOAST classification**			1.330	0.704–2.512	0.380
LAA, n (%)	10 (76.9)	123 (51.5)			
CE, n (%)	0 (0.0)	18 (7.5)			
SAO, n (%)	1 (7.7)	77 (32.2)			
ODC, n (%)	2 (15.4)	5 (2.1)			
UND, n (%)	0 (0.0)	16 (6.7)			

*BG* blood glucose, *BMI* body mass index, *CE* cardioembolism, *DBP* diastolic blood pressure, *DNT* door to needle time, *END* early neurological deterioration, *IQR* interquartile range, *LAA* large-artery atherosclerosis, *NIHSS* National Institute of Health Stroke Scale, *NLR* neutrophil-to-lymphocyte ratio, *ODC* stroke of other determined cause, *OTT* symptom onset to thrombolysis time, *PCS* posterior circulation stroke, *SAO* small-artery occlusion, *SBP* systolic blood pressure, *TOAST* trial of Org 10,172 in acute stroke treatment, *UND* stroke of undetermined cause.

**Figure 3:**
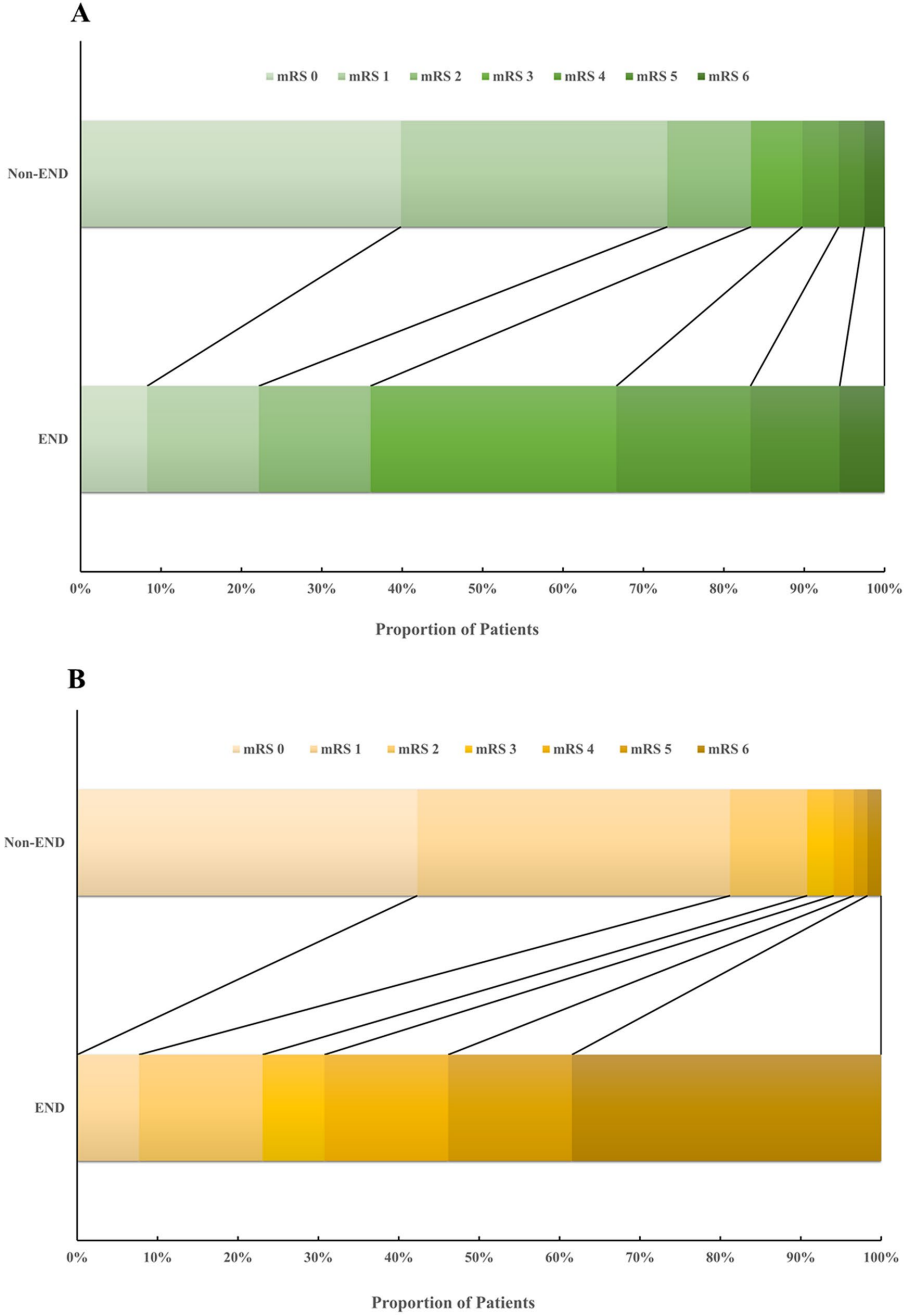
The 90-day mRS distribution in thrombolytic patients with Non-END vs END. (**A**) 90-day mRS distribution in anterior circulation stroke. (**B**) 90-day mRS distribution in posterior circulation stroke. *END* early neurological deterioration, *mRS* modified ranking scale.

Ordinal logistic regression analysis demonstrated that END was associated with worse functional outcomes on the 90-day mRS, compared with Non-END patients in ACS group (OR = 2.301, 95% CI 1.685–2.917, adjusted *p* < 0.001) and PCS group (OR = 3.314, 95% CI 2.132–4.496, adjusted *p* < 0.001), respectively (Fig. 3).

## Discussion

To our best knowledge, this is the first study comparing post-thrombolytic END between ACS and PCS. Our study demonstrated 3 facts: (1) the incidence of END was similar between ACS and PCS; (2) more atrial fibrillation and large-artery atherosclerosis increased risk of END in ACS, while more hypertension history and higher baseline NIHSS score increased risk of END in PCS; (3) END was associated with worse functional outcomes at 90 days in ACS and PCS.

The proportion of patients with PCS in the present study was comparable to that reported in previous studies (21.1% vs 16.8–20.4%)^12,13^. The incidence of END in the present study was lower than that described in a previous meta-analysis (4.1% vs 13.8%)^7^, however similar to that from real-world study (4.1% vs 5.8–6.7%)^8,14^. Longer door-to-needle time caused by delayed neurology evaluation or missed accuracy diagnosis maybe contribute to increased END in PCS^15,16^. Given that symptomatic intracranial hemorrhage was reported as a cause of END, we also investigated the proportion of END due to hemorrhagic injury in two groups. The incidence of hemorrhagic END was similar in ACS vs PCS groups (1.0% vs 1.6%), but lower than that reported in the previous study (2.9%)^17^. The lower proportion of hemorrhagic END could possibly be due to the inclusion of patients with predominantly mild neurological deficits in the present study comparing with previous studies (median baseline NIHSS: 6 vs 15). In agreement with previous studies^7,8^, END was associated with worse functional outcome at 90 days in the present study.

Another highlight of this study was different risk factors of END in ACS vs PCS. In ACS group, we found more atrial fibrillation and large-artery atherosclerosis were associated with END. Consistent with previous findings^8,18^, patients with END tended to have more atrial fibrillation and large-artery atherosclerosis in ACS group, which maybe contribute to poor collateral circulation and ischemic stroke progression^19,20^. Nevertheless, several reported risk factors of END were not found in the present study, such as NIHSS score and blood glucose^7^. These conflicting results may be explained by lower baseline NIHSS score (6 vs 11) and blood glucose (6.7 mmol/L vs 9.0 mmol/L) in the present study comparing with previous studies^8,21^. Unexpectedly, distinct from ACS group, more hypertension history and higher baseline NIHSS score were firstly found to be associated with END in PCS group. Hypertension had been considered as a predictor of END in the previous study^8^, which reduced collateral blood supply and increased risk of post-thrombolytic hemorrhagic transformation^22–24^. Interestingly, NIHSS score seems less effective for evaluating PCS, however it predicted higher risk of END in the present study, which was supported by previous studies finding that NIHSS at admission was associated with END through increasing intracerebral hemorrhage in PCS^25,26^.

### Study limitations

Although this is the first report to find similar incidence, but different risk factors of post-thrombolytic END in ACS vs PCS based on a large-sample, prospective, nationwide registry study, several limitations of our study should be noted. Firstly, uncalculated sample size and large share of excluded patients in this secondary analysis may weaken the power of conclusion. Secondly, as NIHSS seems to be less reliable for PCS, the incidence of END defined with change of NIHSS score may be underestimated in PCS group. And the lower incidence of END in PCS group limited our ability to draw more definitive conclusion. Thirdly, because of lower incidence of END in two groups, we couldn’t comprehensively investigate the specificity and predictive power of risk factors in two stroke territories. Lastly, as INTRECIS study was only performed in Chinese ischemic stroke, the conclusion in the present study had limitation in ethnicity. A cohort with large sample and multiethnic patients warrants further investigation.

## Conclusions

The current study provided the first report about END after intravenous thrombolysis in patients with ACS vs PCS, and found similar incidence, but different risk factors of END in the two distinct types of stroke.

## Methods

### Study population and procedures

The detailed design of INTRECIS study has been reported^10^. From the INTRECIS cohort, patients were included with following criteria: consecutive adult patients (age ≥ 18 years) with brain imaging confirmed AIS who were previously well (modified Rankin Scale [mRS] scores 0 or 1) and received the treatment with 0.9 mg/kg intravenous alteplase (Boehringer Ingelheim Pharma GmbH & Co) within 4.5 h of a definite time of onset of symptoms. Patients were excluded with following criteria: (1) received intravenous thrombolysis with urokinase or non-standard dose of alteplase; (2) no imaging data used to identify stroke territory; (3) received endovascular intervention in 24 h after intravenous thrombolysis; (4) uncompleted baseline clinical data for logistic regression analysis. All patients and/or their legally gave written informed consent for data collection and follow-up.

According to clinical symptom and imaging data, patients were divided into two groups: ACS group, with culprit vessel located in the internal carotid, middle or anterior cerebral artery, and PCS group, with culprit vessel in the vertebral, basilar, or posterior cerebral artery.

We collected baseline characteristics of patients including age, gender, current smoker, current drinker, hypertension, diabetes mellitus, hyperlipidemia, coronary heart disease, atrial fibrillation, history of stroke, body mass index, systolic blood pressure, diastolic blood pressure, symptom onset to thrombolysis time, door to needle time, National Institute of Health Stroke Scale (NIHSS) score, blood glucose, neutrophil-to-lymphocyte ratio, Trial of Org 10,172 in Acute Stroke Treatment (TOAST) classification^11^, and imaging data. Additionally, NIHSS score and imaging data were collected at admission and 24 h after intravenous thrombolysis, respectively. We also collected mRS at 90 days after intravenous thrombolysis.

### Outcomes

The primary outcome was incidence of END after intravenous thrombolysis, which was compared between ACS and PCS groups. END was defined as an increase in NIHSS score ≥ 4 at 24 h, including death within 24 h from baseline^3^. The secondary outcomes were associated factors of END and 90-day mRS distribution.

### Ethics approval

The study was centrally approved by General Hospital of Northern Theater Command Ethics Committee and performed in accordance with the Declaration of Helsinki.

### Statistical analysis

We performed descriptive statistics for baseline characteristics. Continuous variables with abnormal distribution were described as median and inter-quartile range. Continuous variables included age, body mass index, systolic blood pressure, diastolic blood pressure, symptom onset to thrombolysis time, door to needle time, NIHSS scores, neutrophil-to-lymphocyte ratio, and blood glucose. Categorical variables were described as number and proportions. Categorical variables included gender, current smoker, current drinker, hypertension, diabetes mellitus, hyperlipidemia, coronary heart disease, atrial fibrillation, history of stroke, and TOAST classification.

Multivariate logistic regression analysis with adjusting all the baseline variables was used to compare incidence of END between ACS and PCS, and identify factors associated with END. Ordinal logistic regression analysis was used to investigate association between END and 90-day mRS. Results were reported with odds ratios (OR) and 95% confidence intervals (CI). In the relevant analytic tests, differences were considered statistically significant with a *p* value < 0.05. The statistical software SPSS version 23.0 (IBM, NY, USA) was used for the analysis.

## Data Availability

Data are available on reasonable request.

## References

[1] National Institute of Neurological Disorders and Stroke rt-PA Stroke Study Group. Tissue plasminogen activator for acute ischemic stroke. *N. Engl. J. Med.* **333**, 1581–1587 (1995). 10.1056/NEJM199512143332401 7477192

[2] Hacke W, Kaste M, Bluhmki E, Brozman M, Dávalos A, Guidetti D, Larrue V, Lees KR, Medeghri Z, Machnig T (2008). Thrombolysis with alteplase 3 to 4.5 hours after acute ischemic stroke. N. Engl. J. Med..

[3] Sarikaya H, Arnold M, Engelter ST, Lyrer PA, Mattle HP, Georgiadis D, Bonati LH, Fluri F, Fischer U, Findling O, Ballinari P, Baumgartner RW (2011). Outcomes of intravenous thrombolysis in posterior versus anterior circulation stroke. Stroke.

[4] Keselman B, Gdovinová Z, Jatuzis D, Melo TPE, Vilionskis A, Cavallo R, Frol S, Jurak L, Koyuncu B, Nunes AP, Petrone A, Lees KR, Mazya MV (2020). Safety and outcomes of intravenous thrombolysis in posterior versus anterior circulation stroke: Results from the safe implementation of treatments in stroke registry and meta-analysis. Stroke.

[5] Dorňák T, Král M, Hazlinger M, Herzig R, Veverka T, Buřval S, Šaňák D, Zapletalová J, Antalíková K, Kaňovský P (2015). Posterior vs anterior circulation infarction: Demography, outcomes, and frequency of hemorrhage after thrombolysis. Int. J. Stroke..

[6] Sommer P, Posekany A, Serles W, Marko M, Scharer S, Fertl E, Ferrari J, Lang W, Vosko M, Szabo S, Kiechl S, Knoflach M, Greisenegger S (2018). Is functional outcome different in posterior and anterior circulation stroke?. Stroke.

[7] Seners P, Turc G, Oppenheim C, Baron JC (2015). Incidence, causes and predictors of neurological deterioration occurring within 24h following acute ischaemic stroke: A systematic review with pathophysiological implications. J. Neurol. Neurosurg. Psychiatry..

[8] Yu WM, Abdul-Rahim AH, Cameron AC, Kõrv J, Sevcik P, Toni D, Lees KR (2020). The incidence and associated factors of early neurological deterioration after thrombolysis: Results from SITS registry. Stroke.

[9] Seners P, Turc G, Tisserand M, Legrand L, Labeyrie MA, Calvet D, Meder JF, Mas JL, Oppenheim C, Baron JC (2014). Unexplained early neurological deterioration after intravenous thrombolysis: Incidence, predictors, and associated factors. Stroke.

[10] Wang X, Li X, Xu Y, Li R, Yang Q, Zhao Y, Wang F, Sheng B, Wang R, Chen S (2021). Effectiveness of intravenous r-tPA versus UK for acute ischaemic stroke: A nationwide prospective Chinese registry study. Stroke Vasc. Neurol..

[11] Adams HP, Bendixen BH, Kappelle LJ, Biller J, Love BB, Gordon DL, Marsh EE (1993). Classification of subtype of acute ischemic stroke. Definitions for use in a multicenter clinical trial: TOAST Trial of Org 10172 in Acute Stroke Treatment. Stroke.

[12] Sung SF, Chen CH, Chen YW, Tseng MC, Shen HC, Lin HJ (2013). Predicting symptomatic intracerebral hemorrhage after intravenous thrombolysis: Stroke territory as a potential pitfall. J. Neurol. Sci..

[13] Schonewille WJ, Wijman CA, Michel P, Rueckert CM, Weimar C, Mattle HP, Engelter ST, Tanne D, Muir KW, Molina CA (2009). Treatment and outcomes of acute basilar artery occlusion in the Basilar Artery International Cooperation Study (BASICS): A prospective registry study. Lancet Neurol..

[14] Simonsen CZ, Schmitz ML, Madsen MH, Mikkelsen IK, Chandra RV, Leslie-Mazwi T, Andersen G (2016). Early neurological deterioration after thrombolysis: Clinical and imaging predictors. Int. J. Stroke..

[15] Sarraj A, Medrek S, Albright K, Martin-Schild S, Bibars W, Vahidy F, Grotta JC, Savitz SI (2015). Posterior circulation stroke is associated with prolonged door-to-needle time. Int. J. Stroke..

[16] Sato S, Toyoda K, Uehara T, Toratani N, Yokota C, Moriwaki H, Naritomi H, Minematsu K (2008). Baseline NIH Stroke Scale Score predicting outcome in anterior and posterior circulation strokes. Neurology.

[17] Tanaka K, Matsumoto S, Furuta K, Yamada T, Nagano S, Takase KI, Hatano T, Yamasaki R, Kira JI (2020). Differences between predictive factors for early neurological deterioration due to hemorrhagic and ischemic insults following intravenous recombinant tissue plasminogen activator. J. Thromb. Thrombolysis..

[18] Sanák D, Herzig R, Král M, Bártková A, Zapletalová J, Hutyra M, Skoloudík D, Vlachová I, Veverka T, Horák D, Kanovský P (2010). Is atrial fibrillation associated with poor outcome after thrombolysis?. J. Neurol..

[19] Kimura K, Iguchi Y, Shibazaki K, Iwanaga T, Yamashita S, Aoki J (2009). IV t-PA therapy in acute stroke patients with atrial fibrillation. J. Neurol. Sci..

[20] Tisserand M, Seners P, Turc G, Legrand L, Labeyrie MA, Charron S, Méder JF, Mas JL, Oppenheim C, Baron JC (2014). Mechanisms of unexplained neurological deterioration after intravenous thrombolysis. Stroke.

[21] Mori M, Naganuma M, Okada Y, Hasegawa Y, Shiokawa Y, Nakagawara J, Furui E, Kimura K, Yamagami H, Kario K, Okuda S, Koga M, Minematsu K, Toyoda K (2012). Early neurological deterioration within 24 hours after intravenous rt-PA therapy for stroke patients: the stroke acute management with urgent risk factor assessment and improvement rt-PA registry. Cerebrovasc. Dis..

[22] Alvarez FJ, Segura T, Castellanos M, Leira R, Blanco M, Castillo J, Dávalos A, Serena J (2004). Cerebral hemodynamic reserve and early neurologic deterioration in acute ischemic stroke. J. Cereb. Blood Flow Metab..

[23] Toni D, Fiorelli M, Zanette EM, Sacchetti ML, Salerno A, Argentino C, Solaro M, Fieschi C (1998). Early spontaneous improvement and deterioration of ischemic stroke patients: A serial study with transcranial Doppler ultrasonography. Stroke.

[24] Butcher K, Christensen S, Parsons M, De Silva DA, Ebinger M, Levi C, Jeerakathil T, Campbell BC, Barber PA, Bladin C (2010). Postthrombolysis blood pressure elevation is associated with hemorrhagic transformation. Stroke.

[25] Dorňák T, Král M, Sedláčková Z, Šaňák D, Čecháková E, Divišová P, Zapletalová J, Kaňovský P (2018). Predictors for intracranial hemorrhage following intravenous thrombolysis in posterior circulation stroke. Transl Stroke Res..

[26] Saposnik G, Fang J, Kapral MK, Tu JV, Mamdani M, Austin P, Johnston SC (2012). The iScore predicts effectiveness of thrombolytic therapy for acute ischemic stroke. Stroke.

